# Alpha fetoprotein directly induces a unique pro-inflammatory, IL-2 hyperresponsive phenotype in human natural killer cells

**DOI:** 10.1186/2051-1426-2-S3-P178

**Published:** 2014-11-06

**Authors:** Lazar Vujanovic, Elizabeth Stahl, Lisa H Butterfield

**Affiliations:** 1 University of Pittsburgh Cancer Institute, PA, USA; 2 University of Pittsburgh, PA, USA

## 

Alpha fetoprotein (AFP) is an oncofetal antigen commonly produced by hepatocellular carcinomas (HCC). Previous studies have shown that tumor-derived AFP (tAFP) has an immunosuppressive role on natural killer (NK), T, B, and dendritic (DC) cells which may play a role in HCC pathogenesis. Defects in NK cell frequency and function have partially been attributed to tAFP-mediated immunosuppression of DC function. However, a direct tAFP effect on NK cells remains unclear. Here we examine the ability of cord blood-derived AFP (nAFP) and tAFP to modulate human NK cell activity *in vitro*. We show that exposure to tAFP or, especially, nAFP proteins induces a unique pro-inflammatory NK cell activation profile as measured by CD69 upregulation, IL-1β and IL-6 secretion, and enhanced tumor cell killing. Interestingly, AFP-treated plus interleukin-2 (IL-2) stimulated cultures promote a degree of NK cell activation that is higher than that of NK cells activated with IL-2 alone by both phenotypic and functional measures, including elevated IFN-γ and GM-CSF secretion. To confirm that the observed effects are directly mediated by AFP protein, we confirmed that NK cells can readily bind to and take up nAFP and tAFP. The observed synergism between AFP and IL-2 may be mediated by the ability of AFP to modulate IL-2 receptor signaling, as shown by the ability of AFP to upregulate CD25, and downregulate CD122 and CD132 on NK cells. Overall, these data show that nAFP and tAFP induce a unique pro-inflammatory, IL-2 hyperresponive phenotype in NK cells. Defining the impact of circulating AFP on NK cells may be crucial to understand the NK cell functional deficits described in HCC patients, and for the development of an effective HCC-targeting immunotherapy.

